# A case of pervasive refusal syndrome related to COVID19

**DOI:** 10.1192/bjo.2021.333

**Published:** 2021-06-18

**Authors:** Nurul Aqma Mohd Kamil, Sharon Ryan

**Affiliations:** 1 Child & Adolescent Mental Health Service; 2 Mercy University Hospital

## Abstract

**Objective:**

To highlight the importance of appropriate diagnosis and management of severe mental illness in children. Awareness of rare diagnoses such as this will reduce the delay to treatment. A challenge in Ireland is accessing psychiatric inpatient treatment for very young children, with specialist units in Ireland designed to better cater for young people aged 12+.

**Case report:**

Michael (not his real name), age 10, was always described as a happy, calm child. He enjoyed school and loved playing outdoors. He had been progressing well with his life and neither his parents nor school had any concerns for him. Following the COVID-19 pandemic and school closures, Michael began to became more conscious of daily hygiene safety advice. However, things escalated to a very difficult level. Initially, he manifested extreme levels of anxiety with heightened levels of distress. He ran away from open doors or windows for fear he would catch the virus, insisted on changing his clothes several times per day, would become distressed if anyone touched him accidentally while he was outside and could spend hours afterwards crying and screaming.

In June 2020 he showed profound refusal to engage in basic care tasks and a dramatic social withdrawal, and ultimately required admission to hospital. He refused to eat and drink, stopped washing and toileting himself, lay in bed with the covers over this head, became non-verbal and refused to engage with any conversation or games. He showed prolonged periods of screaming. Ultimately this reached a level requiring TPN and PEG feeding and a low stimulation environment. Diagnosis of pervasive refusal disorder, secondary to severe COVID-19 related anxiety was made.

**Discussion:**

Pervasive refusal disorder is a rare and potentially life threatening condition in children. It is described as a profound psychological response to uncontrollable events such as grief, abuse, parental conflict and migration. In this case, it was the threat of the global pandemic. Through treatment in low stimulation environments, with consistent communication and rehabilitation and medication, followed by individual and family therapies when patients are more able, patients show a slow, but generally complete recovery. Happily for Michael, he has now recovered and returned home to his family, where he has returned to all his previous activities.

**Conclusion:**

Michael and his parents have kindly agreed to allow us to tell his story, in the hope of teaching current and future psychiatrists about this rare condition. We send them our thanks and appreciation.

